# The Expression and Activity of Cathepsins D, H and K in Asthmatic Airways

**DOI:** 10.1371/journal.pone.0057245

**Published:** 2013-03-06

**Authors:** Alen Faiz, Gavin Tjin, Louise Harkness, Markus Weckmann, Shisan Bao, Judith L. Black, Brian G. G. Oliver, Janette K. Burgess

**Affiliations:** 1 Cell biology, Woolcock Institute of Medical Research, Sydney, New South Wales, Australia; 2 Central Clinical School, The University of Sydney, Sydney, New South Wales, Australia; 3 Discipline of Pharmacology, The University of Sydney, Sydney, New South Wales, Australia; 4 Discipline of Pathology, School of Medical Science and Bosch Institute, The University of Sydney, Sydney, New South Wales, Australia; 5 Cooperative Research Centre for Asthma and Airways, Sydney, New South Wales, Australia; Gentofte University Hospital, Denmark

## Abstract

Tumstatin is an anti-angiogenic collagen IV α3 fragment, levels of which are reduced in the airways of asthmatics. Its reduction may be due to the degradation by extracellular matrix (ECM) proteases. Cathepsins play a role in ECM remodelling, with cathepsin D, H and K (CTSD, CTSH and CTSK) being associated with lung diseases. CTSD modulates the NC1 domains of collagen molecules including tumstatin, while CTSH and CTSK are involved in ECM degradation. The role of these cathepsins in the regulation of tumstatin in the lung has not previously been examined. We demonstrated that CTSB, D, F, H, K, L and S mRNA was expressed in the airways. Quantification of immunohistochemistry showed that there is no difference in the global expression of CTSD, CTSH and CTSK between asthmatics and non-asthmatics. CTSD and CTSK, but not CTSH had the capacity to degrade tumstatin. No difference was observed in the activity of CTSD and H in bronchoalveolar lavage fluid of asthmatic and non-asthmatics, while CTSK was undetectable. This indicates that while CTSD possesses the potential to directly regulate tumstatin, and thus angiogenesis through this mechanism however, it is not likely to be involved in the dysregulation of tumstatin found in asthmatic airways.

## Introduction

Asthma is a chronic inflammatory disease of the airways which is characterized by airway hyperresponsiveness [1] and airway remodelling [2], [3]. Airway remodelling involves structural changes including increased airway smooth muscle mass, thickening of the basement membrane, increased angiogenesis and altered composition of the extracellular matrix (ECM) [2], [3].

Angiogenesis is the formation of new blood vessels from existing ones. In healthy individuals, this process is tightly regulated by a balance of pro- and anti-angiogenic factors. However in asthmatics, this balance is disrupted and there is an increase in both the number and the size of blood vessels in the airway tissue [4], [5].

The ECM is a complex network of macromolecules which maintains the structural integrity of tissues. In asthmatic airways the composition of the ECM is altered compared to non-asthmatics, with an increased deposition of collagen I, III and V, fibronectin, heparan sulfate proteoglycans and various other proteins, and reduced deposition of elastin and collagen IV (COL4) [6], [7]. COL4 is a major component of the ECM which has an important role in regulating endothelial cell proliferation and other behaviours. The formation and survival of blood vessels is connected with collagen synthesis and deposition in the basement membrane [8], particularly inhibition of collagen metabolism which has anti-angiogenic effects [9].

Six distinct isoforms of COL4 α-chains have been identified (α1–6). The COL4 α-chains have a basic domain configuration consisting of the N-terminal 7S domain, the collagenous domain and the C-terminal globular non-collagenous domain 1 (NC1) [10]. Recently it has been acknowledged that the ECM is a reservoir for endogenous growth factors. In the body, endogenous proteases such as matrix metalloproteinases (MMPs) and their inhibitors tissue inhibitor metalloproteinase (TIMPs) are involved in routine ECM turnover for the maintenance of normal tissue homeostasis. Degradation of COL4 α-chains by MMPs leads to the release of the NC1 domains which can function as active peptides. One example is the NC1 domain of the COL4α3 chain, tumstatin, a potent anti-angiogenic factor [2], [8], [11], [12], [13].

Tumstatin expression was found to be significantly reduced in human asthmatic compared to non-asthmatic airway tissue sections [2]. As COL4α3 is deposited in the ECM as a component of a heterotrimer of α3α4α5 [14] the presence of the other NC1 domains of the COL4 α-chains in the asthmatic airway tissue [2], indicate that the NC1 domain of COL4α3 was likely to have been degraded following deposition into the ECM by the action of endogenous proteases.

One such family of protease is the cathepsins, papain like cysteine and aspartic proteases capable of degrading ECM components with unique collagenolytic activity. They can function both intra- and extracellularly and also participate in wound healing and tumour cell invasion [15], [16]. Cathepsins are expressed in most tissues throughout the body. Cathepsin D (CTSD), H (CTSH) and K (CTSK) are found in the lung and are associated with inflammatory lung diseases [17], whilst other members of the cathepsin family such as cathepsin B (CTSB), F (CTSF), L (CTSL) and S (CTSS) [18] have the potential to participate in ECM remodelling. The expression and activity of these proteases in asthma is unknown.

CTSB, F, H, K, L and S are cysteine proteases which are predominantly expressed by lung granulomatous lesions, bronchial epithelium [19], metastatic lung tumors [20], [21] and inflammatory cells [22], [23], [24], [25], [26], [27]. CTSK is the most potent mammalian elastase reported to date [16], [28]. CTSD is an aspartic protease that digests expired and denatured proteins or abnormal proteins into large fragments which are more accessible by other proteases. It cleaves proteoglycan polypeptides and collagen [29].

A number of cathepsins have previously been identified as having a role in airway diseases. Expression of CTSD is increased in cystic fibrosis [30], and has a role in airway remodelling during fibrogenesis in pulmonary fibrosis [31]. CTSH is upregulated in asthmatic serum [32] and it is increased in the parenchyma of smoking but not non-smoking lung cancer patients [33]. CTSK elicits a protective role during lung matrix homeostasis under physiological and pathological conditions [16], [34]. In addition, CTSK is overexpressed in lymphangioleiomyomatosis (LAM) cells and related renal angiolipomas [17]. Whilst it has yet to be reported in human studies, CTSS is elevated in murine models of allergic asthma and CTSS inhibition is prophylactic to ameliorate airway inflammation [35].

Given the potential of cathepsins to degrade the ECM, and COL4 in particular, the aim of this study was to investigate the role of the highlighted cathepsins in the regulation of tumstatin in the asthmatic airway.

## Results

### Expression of Cathepsins

**Figure 1:**
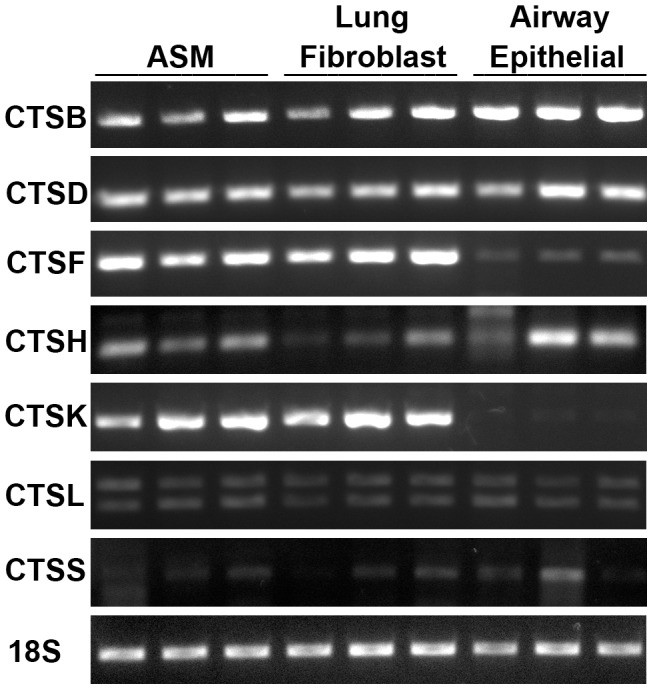
CTSB, D, F, H, K, L and S mRNA expression quantified by reverse transcriptase PCR. ASM (n = 3), lung fibroblast (n = 3) and airway epithelial (n = 3). Predicted band size indicated by (−). Abbreviations CTSB = cathepsin B, CTSD = cathepsin D, CTSF = cathepsin F, CTSH = cathepsin H, CTSK = cathepsin K, CTSL = cathepsin L, CTSS = cathepsin S and ASM = airway smooth muscle.

To investigate the presence of CTSB, D, F, H, K, L and S in human airways, reverse transcriptase-PCR was performed on cells isolated from human lungs to identify mRNA expression. Expression of all cathepsin mRNA was confirmed in primary human airway smooth muscle (ASM), airway epithelium and lung fibroblasts (Figure 1). Two splice variants were found to be expressed by CTSL.

#### Cathepsin protein production and release by structural cells

**Figure 2:**
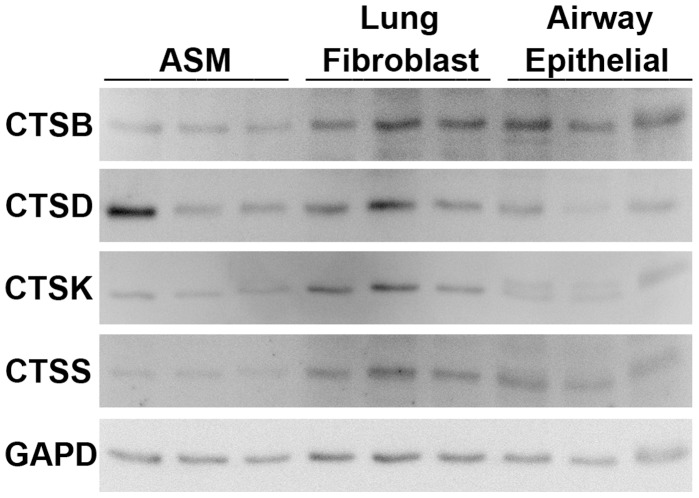
CTSB, D, K and S protein expression quantified by western immunoblot. A representative image of GAPD for each membrane is shown. ASM (n = 3), lung fibroblasts (n = 3) and airway epithelial (n = 3). Abbreviations CTSB = cathepsin B, CTSD = cathepsin D, CTSK = cathepsin K, CTSS = cathepsin S, GAPD = glyceraldehyde-3-phosphate dehydrogenase and ASM = airway smooth muscle.

To investigate the release of cathepsins by structural cells, western immunoblots were conducted on total cell lysates (intracellular cathepsin) and supernatants (extracellular cathepsin) on ASM and lung fibroblasts. Intracellular CTSD, B, K and S presence was identified in all cell types (Figure 2), but no release detected (data not shown). CTSH, L and F were not examined by western blot as the tools for this were not available.

### Detection of Cathepsins in Airway Tissue

**Figure 3:**
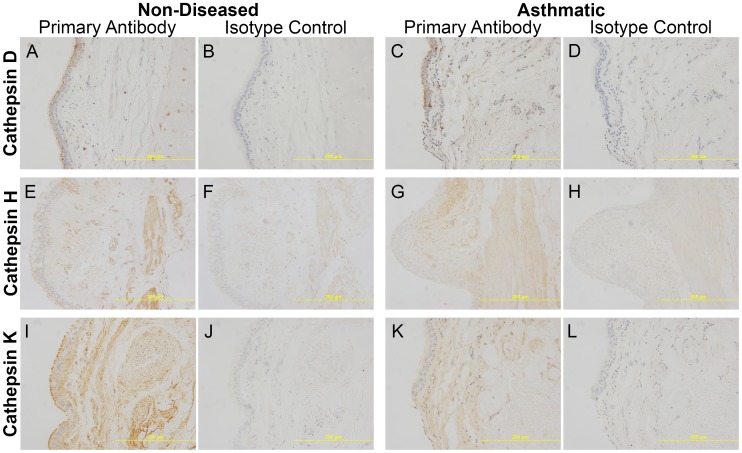
Representative images (20x magnification) of airway sections stained for cathepsins. Immunostaining of cathepsins and corresponding isotype controls for cathepsins D (A–D), H (E–H) and K (I–L) from non-diseased and asthmatic sections. Specific staining was detected using a chemical chromophore DAB (brown) and cell nucleus was counterstained with haematoxylin (blue). Abbreviations DAB = 3,3′-diaminobenzidine.

**Figure 4:**
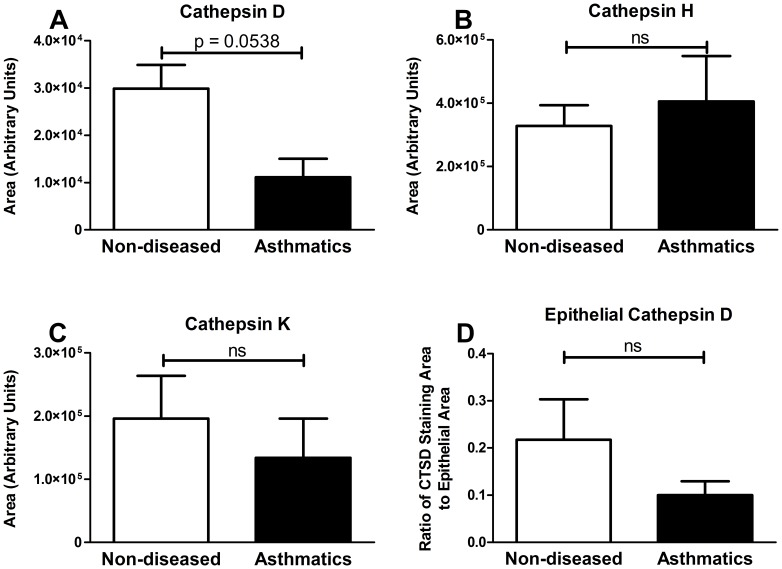
Cathepsin D, H and K expression in non-diseased and asthmatic tissue. Area of cathepsin D (A, ND = 15 and A = 5), cathepsin H (B, ND = 13 and A = 5), cathepsin K (C, ND = 15 and A = 6) and epithelial specific cathepsin D (D, ND = 12 and A = 5) immunostaining in non-diseased and asthmatic sections were quantified using computerized image analysis. Data are expressed as mean ± standard error of the mean. Statistical analysis used was student’s t-test. Abbreviations A = Asthmatic, ND = Non-Diseased and ns = no significance.

Immunohistochemistry (IHC) was then performed on paraffin embedded formalin fixed airway tissue sections to identify protein production of CTSD, CTSH and CTSK. IHC showed that the three cathepsins were expressed in the airways; CTSH and CTSK were ubiquitously expressed, while CTSD appeared to have high expression confined to the epithelial layer with underlying low expression throughout the tissue (Figure 3). Densitometric analysis comparing asthmatic and non-diseased control sections showed no difference in the staining of CTSD, CTSH and CTSK (Figure 4a, b, c). As CTSD expression was localised to the epithelial layer, to avoid bias due to epithelial layer variability in each tissue section densitometry specifically targeting the epithelial layer was performed and the results normalized to the area of epithelial layer analyzed, which detected no difference between asthmatic and non-asthmatics (Figure 4d).

#### Recombinant tumstatin digestion by cathepsins

**Figure 5:**
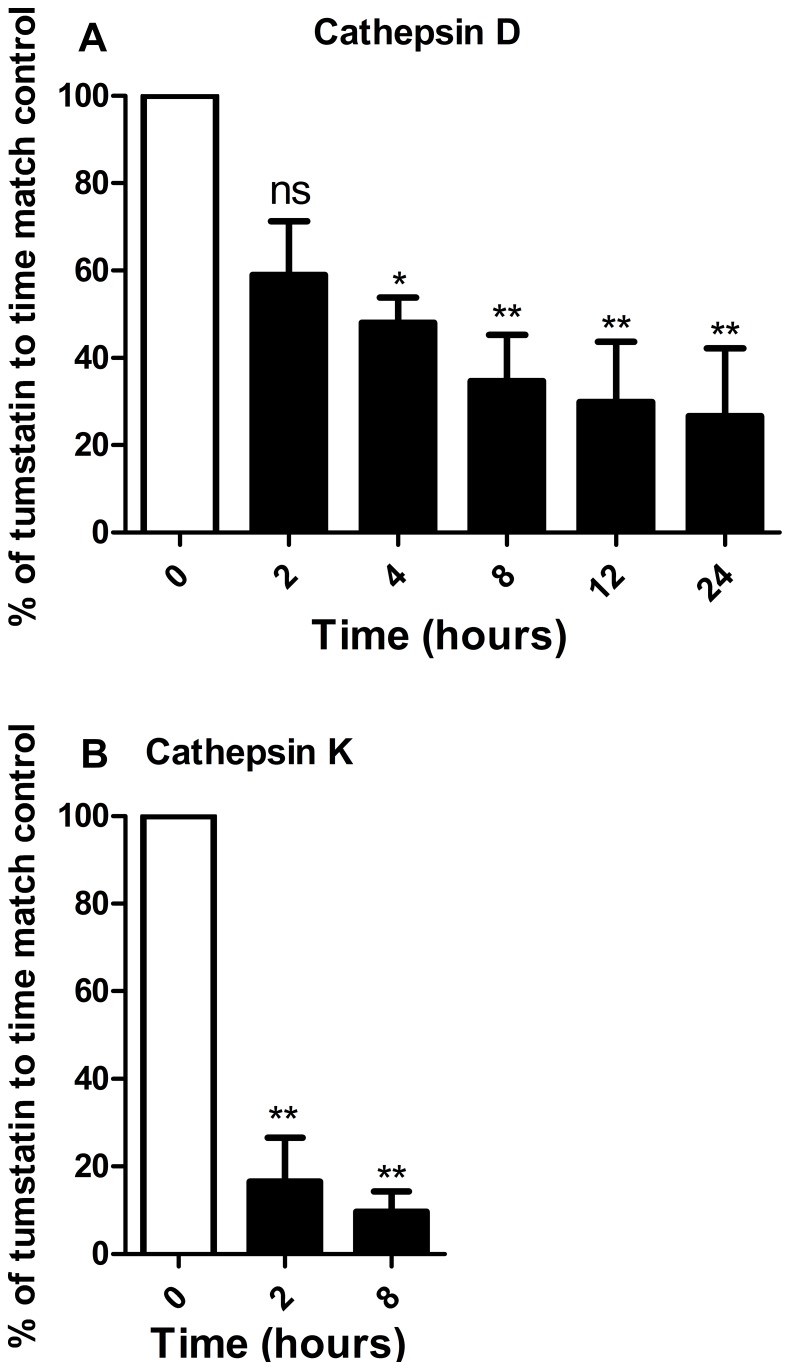
Tumstation digestion by cathepsins. Digestion assay was performed using recombinant human tumstatin (0.1 mg/ml) and cathepsin D (0.028 µM) 0, 2, 4, 8, 12 and 24 hours (n = 3) or cathepsin K (1.88 µM) 0, 2 and 8 hours (n = 3), visualized by western immunoblot and quantified by densitometry. Statistical analysis used was one-way ANOVA. Data are expressed as mean ± standard error of the mean. Abbreviations ns = no significance, * = p<0.05 and ** = p<0.01.

A digestion assay was conducted to evaluate the ability of CTSD, CTSH and CTSK to degrade recombinant human tumstatin using purified enzymes at close to physiological concentrations [36], [37]. The physiological concentration of CTSK in human serum is at 3.1–9.4 pM [38], [39] however the levels of CTSK in the lung were unknown; as such the concentration used for CTSH was used as a guide. These concentrations may not represent the localized extracellular matrix concentrations in tissue where much higher concentrations can be achieved locally following release from lysosomes. CTSD and CTSK digestion of recombinant tumstatin occurred significantly at 4 and 2 hours, respectively (Figure 5), while no digestion was observed with CTSH (data not shown).

#### Cathepsin activity in bronchoalveolar lavage fluid (BALF)

**Figure 6:**
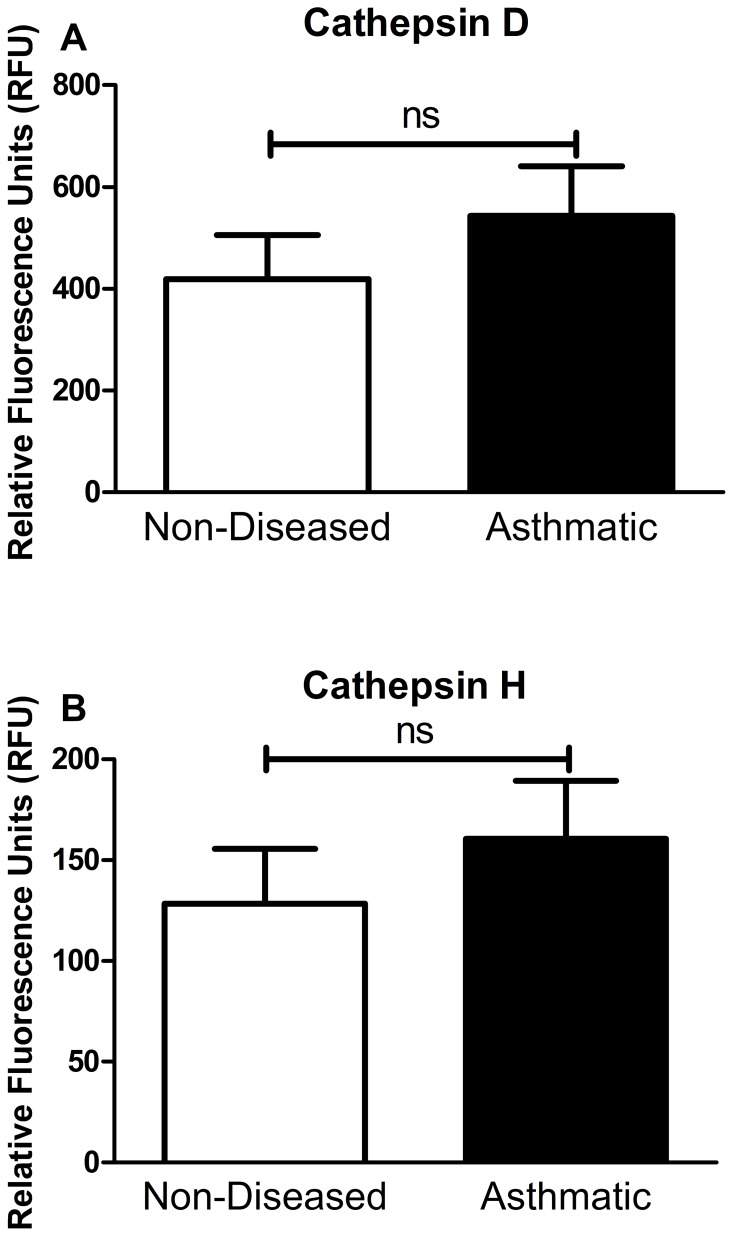
Cathepsin D and H activity assay of BALF. Cathepsin D (A) and cathepsin H (B) activity assay graphs comparing asthmatic (n = 6) and non-asthmatic (n = 8) BALF samples after 2 hours of incubation in reaction buffer. Statistical analysis used was student’s t-test. Data are expressed as mean ± standard error of the mean. Abbreviations ns = no significance and BALF = bronchoalveolar lavage fluid.

There was no difference between CTSD or CTSH activity measured between asthmatic and non-asthmatic BALF (Figure 6) while there was no measureable CTSK activity in asthmatic and non-asthmatic BALF (data not shown).

## Discussion

In this study we have shown, for the first time, that airway structural cells express CTSB, D, F, H, K, L and S mRNA and protein, except for CTSH, F and L for which the protein was not examined. CTSD, H and K were also detected in airway tissues from people with and without asthma. Purified CTSD and K, but not H, were able to digest recombinant tumstatin, however there were no differences in the activity of CTSD or CTSH in BALF from people with and without asthma.

Quantification of the immunohistochemistry results showed that there were no differences in the global expression of CTSD, CTSH and CTSK between asthmatics and non-asthmatics. Due to a potential confounder caused by epithelial shedding which is a process that occurs in asthmatic airways, analysis of CTSD immunostaining was repeated to analyze specifically the differences on the epithelial layer. The results showed that there was no significant difference between the two patient groups. However, the immunostaining showed both intra- and extracellular expression of the enzymes and not the secreted enzymes which would act on the ECM and thus tumstatin. The proteolytic activity of cathepsins is also regulated by endogenous protease inhibitors such as cystatins and while no difference was detected in the protein expression there may still be differences in the proteolytic activity.

The potential of CTSD, CTSH and CTSK to degrade tumstatin was investigated which revealed that CTSD and CTSK but not CTSH have the ability to degrade tumstatin. CTSK activity was not detectable in the BALF and it is likely that this is caused by auto-degradation of CTSK enzyme during storage [40]. However there was no difference observed in the activity of CTSD in BALF of asthmatic and non-asthmatics. This indicates that while CTSD possess the potential to directly regulate tumstatin, and thus angiogenesis through this mechanism, it is not likely to be involved in the dysregulation of tumstatin found in asthmatic airways. However CTSD may indirectly affect tumstatin regulation through the ability to activate or inactivate other proteases and as such the CTSD in the asthmatic airway could lead to a net increase in proteolytic activity. One of the indirect mechanisms of tumstatin regulation by CTSD is the conversion of procathepsin B into cathepsin B (CTSB), an important angiogenesis modulator which regulates vascular endothelial growth factor activity in endothelial cells and digests COL4 to release tumstatin [28]. The presence of CTSB mRNA and protein in the airway structural cells examined in this study indicate that this potential mechanism of tumstatin degradation is worth future investigation. There are many other potential proteases, with CTSB, F, L and S identified in this study being candidates, which may be implicated in the dysregulation of tumstatin in asthmatic airways.

## Experimental Procedures

### Human Tissues

Isolated human airway tissue was obtained from explanted and resected lungs and post mortem organ donors with ethical approval from The University of Sydney and participating hospitals (Concord Repatriation General Hospital, Sydney South West Area Health Service and Royal Prince Alfred Hospital) for sample collection. All patients provided written informed consent or in the case of post mortem samples consent was obtained from the next of kin. The samples used as non-diseased controls were from healthy organ donors whose lungs were deemed unfit for use in a transplant procedure. Further information on the patients can be found in Table S1.

### Human Bronchoalveolar Lavage Fluid

BALF samples were collected from asthmatic volunteers in a previous study [41] along with non-asthmatic volunteers. The samples were collected according to the protocol described previously [41]. Briefly, specimens were collected via normal saline lavage of the segmental airways and alveolar spaces (bronchoalveolar lavage). To remove mucus and cells, the BALF was filtered through sterile gauze and centrifuged at 580 g for 5 min. These samples were stored at −80°C for future investigation. Further details on patient demographics can be found in Table S1.

### Immunohistochemical Sample Preparation

Airway tissues were fixed in 4% phosphate-buffered formalin (pH 7.2) and embedded in paraffin at the Histopathology Laboratory at The University of Sydney and archived. Sections of 3 µm thickness were cut using a Shandon Finesse 325 (Thermo Scientific, Waltham, MA) microtome in the Histopathology Laboratory and mounted on Superfrost Plus microscope slides (Lomb Scientific, Taren Point, AUS) to be used for immunohistochemical analysis.

### Immunohistochemistry

Briefly, sections were de-paraffinized and subjected to appropriate antigen retrieval for antibody staining (details in Table S2). Sections were then treated to minimize non-specific background staining and incubated with primary antibodies, goat anti-human CTSD (R&D Systems, Minneapolis, USA) [0.05 µg/mL], polyclonal mouse anti-human CTSH (Abnova, Taipei, Taiwan) [0.625 µg/mL] and CTSK (Abcam, Cambridge, UK) [1 µg/mL]. Tissue staining was then visualized with substrate chromogen, liquid 3,3′-diaminobenzidine (DAB) (DakoCytomation, Glostrup, CA). Images were then taken and immunostaining was quantified using Image Pro Plus version 7 Software (MediaCybernetics, Discovery Way, Acton, MA) [42], [43]. Further details can be found in Appendix S1.

### Tissue Isolation and Culture

Isolation and culture of ASM and lung fibroblasts was carried out according to the methods described previously [44], [45].

The bronchial epithelial cells from were grown separately in Ham’s F-12 (Invitrogen) with the following growth supplements: epidermal growth factor (EGF) 0.5 ng/ml, bovine pituitary extract 50 µg/ml, hydrocortisone 0.5 µg/ml, epinephrine 0.5 µg/ml, transferrin 10 µg/ml, insulin 5 µg/ml, retinoic acid 0.1 ng/ml, triiodothyronine 6.5 ng/ml (Sigma, St. Louis, MO), 1% antibiotics (Invitrogen). Ham’s F-12 (Invitrogen) with added growth factors was used for maintaining and expanding the cells. 0.03% trypsin with 0.3 mM EDTA (GIBCO, Grand Island, NY) was used for dissociation of the cells from culture flasks or plates.

### Lung Fibroblast ASM and Epithelial Cell Culture

Culture of ASM and lung fibroblasts was carried out according to the method described previously [44]. Briefly both ASM and lung fibroblast cells were grown to passage 3–6 in growth media. Cells were plated up in 6 well plate at density 1×10^4^ cell/cm^2^. Cells were then left for 3 days and media was changed to quiescing media DMEM (Invitrogen) supplemented with 0.1% bovine serum albumin (BSA) (Sigma Aldrich, St Louis, MO, USA), and 1% antibiotics (Invitrogen). Protein, mRNA and supernatant samples were collected after 24 hours and stored at −80°C.

Culture of bronchial epithelial cells was carried out according to the method described previously [46]. Bronchial epithelial cells were detached and plated in bronchial epithelial growth medium bronchial epithelial cell growth medium (BEGM; Cambrex Bio Science, Walkersville, MD) and grown on 12 well cell culture plates (BD Biosciences). Once cells reached confluence (3 days), medium was changed to quiescing medium, bronchial epithelial basal medium (BEBM). Supernatants, cellular protein and RNA lysate were collected at 24 hours and stored at −80°C.

### mRNA Extraction and Conversion

mRNA was extracted using the NucleoSpin® RNA II kit, according to manufacturer’s instructions (Macherey Nagel, Duren, Germany). Quality and quantity of mRNA was determined using a Nanodrop 1000 (NanoDrop Technologies, Inc. Wilmington, DE, USA). 200 ng of mRNA was converted to cDNA with the MMLV reverse transcriptase (Invitrogen) according to the manufacturer’s instructions. Samples were stored at −20°C before use.

### Reverse Transcriptase Polymerase Chain Reaction (RT-PCR)

Primers were designed spaning exon-exon junctions (Geneworks, SA, AUS). RT-PCR reactions contained 5 µM forward primer and reverse primer; 0.2 µM of each dGTP, dCTP, dATP and dTTP; 2 mM MgCl_2_; 10×NH_4_ buffer (Bioline, London, UK); 2.5 units BIOTAQ DNA polymerase (Bioline) and 2 µl cDNA. Total reaction volume was made up to 25 µl. PCR amplifications were conducted on an Eppendorf gradient PCR machine (Eppendorf, Hamburg, Germany) with conditions:−95°C for 2 min, (95°C for 1 min 55–60°C for 1 min and 72°C for 1 min)×25–35 cycles (Table S3).

### Western Immunoblot

Protein lysates and supernatants were run on 10% polyacrylamide gels and transferred to polyvinylidene difluoride membranes (Millipore) and block for 1 hour in 5% BSA. The membranes were washed then incubated overnight with primary antibody (rabbit anti-human COL4α3 Pro-1426 1:20000 (Assaybiotech, California, USA), mouse anti-human CTSB 1∶400 (Abcam), goat anti-human CTSD 1∶500 (R&D), rabbit anti-human CTSK 1∶1000 (Abcam), goat anti-human CTSS 1∶200 (Abcam) or mouse anti-human glyceraldehydes-3-phosphate dehydrogenase (GAPD) 1∶20000 (Millipore) in 1% BSA. The membranes were washed then incubated for 1 hour with appropriate secondary antibody (Dako) in 1% BSA. Detection was performed using Immobilon Western (Millipore) and bands were analyzed using a Kodak imaging system and software. Membranes were stripped and reprobed for as a loading control GAPD or other cathepsins.

### Cloning of Tumstatin and Expression in Escherichia coli (E.coli)

Primers were designed for the N and C terminal regions of COL IV α 3 NC1 (derived from Uniprot, code Q01955; ∼28 kDa; 225aa) according to [47]. To obtain COL IV α 3 mRNA, endothelilal cells were extracted from pulmonary blood vessel dissections of donor lungs as described earlier [2]. These cells were grown to confluence and total RNA was extracted using NucleoSpin RNA II kit according to manufacturer’s instructions (Macherey Nagel, Düren, Germany). Total RNA was then transcribed into cDNA using hexameric primers (New England Biolabs, Ipswich, MA, USA) and Superscript III (Invitrogen). cDNA then was amplified with the following primers: Forward: 3′-GGAATTCCATATGCCGGGTTTGAAAGGAAAACGTC-5′, 3′-CGGGATCCTCAGTGTCTTTTCTTCATGCA-5′ (reverse) with restriction sites for NdeI (forward) and BamHI (reverse): PCR amplification was undertaken for 35 cycles with the following conditions denaturation at 95°C for 15 sec, annealing at 60°C for 30 sec and elongation at 72°C for 60 sec. The amplicon (750 bp) was eluted from a 1.5% Agarose gel (Amresco, Cochran Solon, OH, USA) using a QIAEX II gel extraction kit (Qiagen, Doncaster, VIC, Australia) and cloned into pcDNΑ5/FRT/TO-TOPO (Invitrogen) according to manufacturer’s recommendations. The vector was then transformed into TOP10 E.coli (Invitrogen) and streaked on agar plates with ampicillin (100 µg/mL) (Sigma, St. Louis, MO, USA). Colonies were picked, expanded and the inserts within the isolated plasmids were subject to sequencing (Supamac, Sydney, Australia). Positive clones were selected and archived for later use. Tumstatin was then subcloned into pET15b (via BamHI and NdeI) and transformed into BL21 (DE3) (Bioline, Sydney, NSW, Australia) for expression. E.coli were grown overnight, and then expansion cultures were started with an innoculum of OD 0.1 and grown until they reached OD 0.5. Expression was then induced with 500 µM of isopropyl 1-thio-β-D-galactopyranoside (IPTG, Sigma, St. Louis, MO, USA) for 4 h and cells were pelleted thereafter at 4°C at 4000×g for 40 min. Pellets were collected and then resuspended in buffer A (50 mM Tris–HCl, 5 mM EDTA, pH 7.5). Cells were then sonicated on ice for 50 cycles (4s at 60% of max. amplitude and 6 s pause). The suspension was pelleted at 15 000×g for 20 min before washing with solubilisation buffer 1 (1% Triton X-100 and 3 M urea). The supernatant (15000×g, 20 min) was removed and inclusion bodies were incubated with solubilisation buffer 2 (6 M guanidine hydrochloride, 0.1 M NaH_2_PO_3_, and 10 mM Tris–HCl, pH 5.5) for 2 h at RT. Insoluble debris was spun down and the lysate was either purified via a Nickel-sepharose column (AmershamPharmacia, GE Healthcare, Rydalmere NSW, Australia) or directly processed by dilution and dialysis. For dilution and dialysis, 10 mM dithiothreitol (DTT) was added to the inclusion body lysis solution (IBS) and incubated for 15 min at RT. IBS was then diluted and concentrated twice as follows: Dilution with MilliQ water and centrifugation at 1000 rpm for 45 min at 4°C in ultra filtration units (Amicon Ultra15, 10 kDa, (Millipore). Final concentrate was dialysed against MilliQ water (1∶4000). Purified protein was analysed on polyacrylamide gel electrophoresis (PAGE) for purity (Coomassie Blue staining) and frozen at −80°C for later use. The protein concentration was measured by UV (280 nm, NanoDrop, Wilmington, DE, USA) and Bicinchoninic Acid Assay (Sigma).

### Enzyme Digestion Assay

Enzyme digestion assays were performed on recombinant human tumstatin using purified CTSD (Sigma), CTSH (Enzo Life Sciences, Farmingdale, USA) and pro-cathepsin K (Enzo Life Sciences). Pro-cathepsin K was activated according to manufacturer’s instructions by adding 32.5 mM sodium acetate pH 3.5 to pro-cathepsin K at 1∶6 ratio and incubation at room temperature for 3 hours. Cathepsins were used to digest 0.1 mg/mL tumstatin at concentrations within the active range previously identified in the literature. CTSD digestion was performed at 27.5 nM CTSD in 200 mM sodium citrate at pH 5.0 [48]. CTSH digestion was performed at 1 mM CTSH in 0.05 mM sodium acetate, 10 mM ethylenediaminetetraacetic acid (EDTA) and 1 mM L-cysteine HCL Monohydrate at pH 5.5 [49]. CTSK digestion was performed at 1.83 mM CTSK in 50 mM sodium acetate, 2 mM DTT, 2 mM EDTA acid and 350 mM sodium chloride at pH 7.0 [50]. Digestion experiments were performed over a time course of 0, 2, 4, 8, 12 and 24 hours. The reactions were terminated by the addition of loading dye 0.312 M tris-HCl, 50% glycerol, 10% SDS, 0.5 M DTT and 0.05% bromophenol blue at pH 6.8 to 1∶5 ratio and heat inactivation for 10 mins at 95°C. Samples were visualised by western immunoblot using an anti-tumstatin antibody (Assay Biotechnology, Sunnyvale USA) [40 ng/mL].

### Cathepsin D, H and K Activity Assay

Activity of the enzymes was analysed using fluorometric activity assay kits for cathepsin D (Abcam), H (Abcam) and K (Abcam). Serum and BALF samples were diluted 1∶1 with lysis buffer (triton X-100 1% (w/v)) and loaded onto a 96-well black with clear bottom plate (BD Biosciences). Then an equal volume of mastermix containing reaction buffer and fluorometric substrate was added into each assay well. Positive control proteins cathepsin D (Sigma-Aldrich) [500 ng/well], H (Enzo Life Sciences) and K (Enzo Life Sciences) were added and a negative control of lysis buffer was also included. All assay samples were measured in duplicate.

## Supporting Information

Table S1 **Patient demographics of the individual patients within the sample group used for immunohistochemical analysis and enzyme activity assays.**
(DOCX)

Table S2 **Antibody dependent treatment of airway sections.**
(DOCX)

Table S3 **PCR conditions for cathepsin primers.**
(DOCX)

Appendix S1 **Details of immunohistochemistry and image analysis.**
(DOCX)
