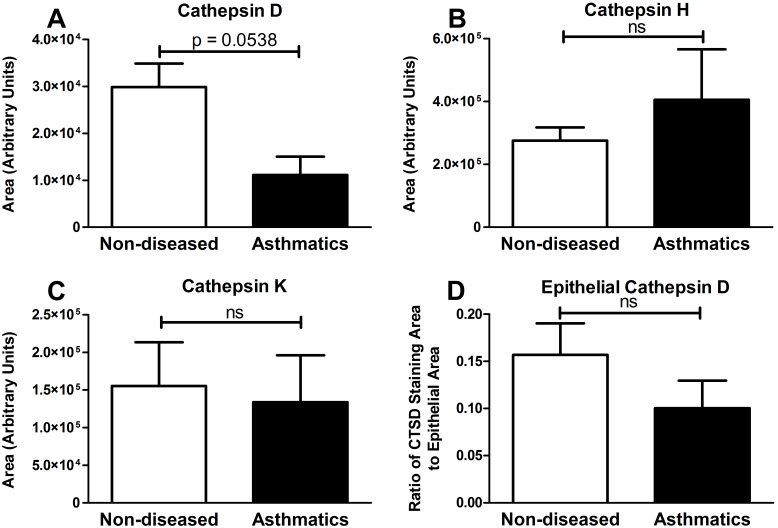# Correction: The Expression and Activity of Cathepsins D, H and K in Asthmatic Airways

**DOI:** 10.1371/annotation/d2be9c62-8d74-4cfb-b49c-b900ea8cd17c

**Published:** 2014-01-14

**Authors:** Alen Faiz, Gavin Tjin, Louise Harkness, Markus Weckmann, Shisan Bao, Judith L. Black, Brian G. G. Oliver, Janette K. Burgess

An error was introduced in the preparation of this article for publication. The scale and unit in panels C and D of Figure 4 are incorrect. Please see the corrected Figure 4 here: 

**Figure:**