# Supplementary material for: The Expression and Activity of Cathepsins D, H and K in Asthmatic Airways
Source: PLoS One. 2013 Mar 6;8(3):e57245. doi: 10.1371/journal.pone.0057245 (PMC3590183; doi:10.1371/journal.pone.0057245)
Supplement: Table S1 — Patient demographics of the individual patients within the sample group used for immunohistochemical analysis and enzyme activity assays. (DOCX) [file pone.0057245.s001.docx]

Table S1. Patient demographics of the individual patients within the sample group used for immunohistochemical analysis and enzyme activity assays.

| No. | Diagnosis | Age | Gender | Samples | Experiments where used |
| --- | --- | --- | --- | --- | --- |
| 1 | Donor - Haemorrhage | 30 | M | Explanted Lung | IHC (D, H and K) |
| 2 | Donor - Haemorrhage | 53 | M | Explanted Lung | IHC (D, H and K) |
| 3 | Donor – Trauma | 40 | M | Explanted Lung | IHC (D, H and K) |
| 4 | Donor – MVA | 19 | M | Explanted Lung | IHC (D, H and K) |
| 5 | Donor – Trauma | 33 | M | Explanted Lung | IHC (D, H and K) |
| 6 | Donor - Haemorrhage | 50 | M | Explanted Lung | IHC (D and K) |
| 7 | Donor - Trauma | 19 | M | Explanted Lung | IHC (D and K) |
| 8 | Donor - Haemorrhage | 52 | M | Explanted Lung | IHC (D, H and K) |
| 9 | Donor - Trauma | 22 | M | Explanted Lung | IHC (D, H and K) |
| 10 | Donor - MVA | 30 | M | Explanted Lung | IHC (D, H and K) |
| 11 | Donor - MVA | 25 | M | Explanted Lung | IHC (D, H and K) |
| 12 | Donor - MVA | 47 | M | Explanted Lung | IHC (D, H and K) |
| 13 | Donor - Trauma | NA | NA | Explanted Lung | IHC (D, H and K) |
| 14 | Donor - MVA | 16 | M | Explanted Lung | IHC (D, H and K) |
| 15 | Donor | NA | NA | Explanted Lung | IHC (D, H and K) |
| 16 | Donor | 52 | M | NA | IHC (D, H and K) |
| 17 | Donor | 45 | F | Explanted Lung | IHC (D) |
| 18 | α1AD Asthma | 39 | M | Explanted Lung | IHC (D, H and K) |
| 19 | Eisenmenger's Asthma | 17 | M | Explanted Lung | IHC (D, H and K) |
| 20 | PPHT Asthma | 33 | M | Explanted Lung | IHC (D and K) |
| 21 | Asthma | 15 | M | Status Asthmaticus | IHC (D, H and K) |
| 22 | Asthma | 80 | M | Donor | IHC (D, H and K) |
| 23 | Asthma | 13 | F | Post Mortem | IHC (H and K) |
| 24 | Normal Volunteer | 27 | F | BALF | CTS AA |
| 25 | Normal Volunteer | 19 | M | BALF | CTS AA |
| 26 | Normal Volunteer | 20 | M | BALF | CTS AA |
| 27 | Normal Volunteer | 31 | M | BALF | CTS AA |
| 28 | Normal Volunteer | 22 | F | BALF | CTS AA |
| 29 | Normal Volunteer | 22 | M | BALF | CTS AA |
| 30 | Asthmatic Volunteer | 18 | F | BALF | CTS AA |
| 31 | Asthmatic Volunteer | 20 | F | BALF | CTS AA |
| 32 | Asthmatic Volunteer | 19 | M | BALF | CTS AA |
| 33 | Asthmatic Volunteer | 20 | M | BALF | CTS AA |
| 34 | Asthmatic Volunteer | 19 | F | BALF | CTS AA |
| 35 | Asthmatic Volunteer | 25 | M | BALF | CTS AA |
| 36 | Asthmatic Volunteer | 21 | M | BALF | CTS AA |
| 37 | Asthmatic Volunteer | 29 | F | BALF | CTS AA |
| 38 | Asthmatic Volunteer | 25 | M | BALF | CTS AA |
| 39 | Donor-MVA | 16 | M | Explanted Lung (ASM) | W, RT-PCR |
| 40 | Normal Volunteer | 69 | M | Biopsy (ASM) | W, RT-PCR |
| 41 | Normal Volunteer | 22 | M | Biopsy (ASM) | W, RT-PCR |
| 42 | NSCCa | 73 | F | Resection (Lung Fibroblast) | W, RT-PCR |
| 43 | Acute interstial pneumonia | 38 | M | Explanted Lung (Lung Fibroblast) | W, RT-PCR |
| 44 | COPD, Carcinoma | 63 | M | Resection (Lung Fibroblast) | W, RT-PCR |
| 45 | Idiopathic Pulmonary Fibrosis | 45 | M | Explanted Lung (Airway epithelial) | W, RT-PCR |
| 46 | Carcinoma | 77 | F | Resection (Airway epithelial) | W, RT-PCR |
| 47 | Emphysema | 64 | M | Resection (Airway epithelial) | W |
| 48 | COPD | 63 | M | Explanted Lung (Airway epithelial) | RT-PCR |

^Abbreviations used M = male, F = female, NA = not available, IHC = immunohistochemistry, D = cathepsin D, H = cathepsin H, K = cathepsin K, COPD=^ ^Chronic obstructive pulmonary disease, MVA = motor vehicle accident, α1AD = α1 antitrypsin deficiency, NCCCa= Non-Small Cell Carcinoma PPHT = primary pulmonary hypertension, BALF = bronchoalveolar lavage fluid, CTS AA = cathepsin activity assays, W= Western Immunoblot, RT-PCR= Reverse Transcriptase Polymerase Chain Reaction^
