# Supplementary material for: The Expression and Activity of Cathepsins D, H and K in Asthmatic Airways
Source: PLoS One. 2013 Mar 6;8(3):e57245. doi: 10.1371/journal.pone.0057245 (PMC3590183; doi:10.1371/journal.pone.0057245)
Supplement: Table S2 — Antibody dependent treatment of airway sections. (DOCX) [file pone.0057245.s002.docx]

Table S2. Antibody dependent treatment of airway sections.

| Antibody | Antigen Retrieval | Non-immune blocking serum |
| --- | --- | --- |
| Goat anti- CTSD | Sodium Citrate Tween-20 pH 6.0 solution at 60^o^C for 2 hours | 1.5% Normal Rabbit Serum |
| Mouse anti- CTSH | Sodium Citrate Tween-20 pH 6.0 solution at 60^o^C for 2 hours | 10% Normal Horse Serum |
| Rabbit anti- CTSK | Tris-EDTA pH 9.0 solution at 60^o^C for 2 hours | 10% Normal Horse Serum |

^Abbreviations used CTSD = cathepsin D, CTSH = cathepsin H, CTSK = cathepsin K and EDTA = ethylenediaminetetraacetic acid.^
