# Supplementary material for: The Expression and Activity of Cathepsins D, H and K in Asthmatic Airways
Source: PLoS One. 2013 Mar 6;8(3):e57245. doi: 10.1371/journal.pone.0057245 (PMC3590183; doi:10.1371/journal.pone.0057245)
Supplement: Table S3 — PCR conditions for cathepsin primers. (DOCX) [file pone.0057245.s003.docx]

Table S3. PCR conditions for cathepsin primers.

| Gene | Forward (5’->3’) | Reverse (5’->3’) | T_m_ | Cycles | Size (bp) |
| --- | --- | --- | --- | --- | --- |
| CTSH | AAAGATGCTGTCCTTGGCGG | CGATGGCCTTTCCAGGTTGG | 63 | 30 | 191 |
| CTSK | GGACATGACCAGTGAAGAGGTGG | TAGCTGCCTTGCCTGTTGGG | 63 | 29 | 422 |
| CTSD | TGCTGGACATCGCTTGCTGGAT | TGCCATCGAACTTGGCTGCGA | 63 | 30 | 258 |
| CTSL | CGAACTCTGCTGGCCTTGA | CCACACTGACCCTGATTCTTC | 63 | 30 | 437, 527 |
| CTSS | AAGGGCTCTTCTTGATGGCT | TCTTCACTGGTCATGTCTCCCA | 63 | 33 | 518 |
| CTSB | GCGCTGGGTGGATCTAGGAT | GGAGGGATGGAGTACGGTCT | 63 | 27 | 589 |
| CTSF | TGTCCAAGAAAACCCTGCTCT | CTGGATCTTCTGTGCTCGCA | 63 | 30 | 371 |
| 18S | TCAAGAACGAAAGTCGGAGGTT | GGACATCTAAGGGCATCACAG | 63 | 22 | 489 |

^Abbreviations used CTSD = cathepsin D, CTSH = cathepsin H, CTSK = cathepsin K, CTSL = cathepsin L, CTSS = cathepsin S, CTSB = cathepsin B, CTSF = cathepsin F, bp = base pair and tm = melting temperature.^
