# Supplementary material for: The Expression and Activity of Cathepsins D, H and K in Asthmatic Airways
Source: PLoS One. 2013 Mar 6;8(3):e57245. doi: 10.1371/journal.pone.0057245 (PMC3590183; doi:10.1371/journal.pone.0057245)
Supplement: Appendix S1 — Details of immunohistochemistry and image analysis. (DOCX) [file pone.0057245.s004.docx]

Appendix S1. Details of immunohistochemistry and image analysis.

Sections were de-paraffinized by incubation in xylene (MP Biomedicals, Santa Ana, CA) overnight, then for 10 mins in a change of xylene and rehydrated through graded alcohol (2x 100%, 95% and 70% ethanol) for 2 mins each before being placed in distilled water for at least 2 mins. Sections were subjected to antigen retrieval appropriate for the antibody staining and then blocked with a peroxidase blocking agent (DakoCytomation) for 30 mins before washing three times with tris buffered saline (TBS) for 5 mins each and blocking with the appropriate non-immune blocking serum for 30 mins (details in

Supplement 2). Sections were incubated with primary antibodies, goat anti-human CTSD (R&D Systems) [0.05µg/mL] and polyclonal mouse anti-human CTSH (Abnova) [0.625µg/mL] overnight at 4^o^C in a humid box and the sections with rabbit anti-human CTSK (Abcam) [1µg/mL] were incubated with the antibody for 3 hours at room temperature. Isotype controls were also performed in parallel with the primary antibody immunostaining using purified goat IgG (Zymed, San Francisco, CA), mouse serum (DakoCytomation) and rabbit IgG (DakoCytomation) as appropriate at the same concentration as the primary antibody. Sections were washed five times in TBS with 0.5% Tween-20 (TBS-T) (3 mins each) followed by incubation with the secondary antibodies anti-mouse horseradish peroxidase (HRP) (DakoCytomation) or anti-rabbit HRP (DakoCytomation) at room temperature for 45 mins. For the detection of goat antibodies, rabbit anti-goat biotinylated antibody (Vector Laboratories, Burlingame, CA, USA) was added and incubated for 30 mins at room temperature. These sections were then washed three times in TBS-T for 5 mins each and incubated with AB reagent (Vector Laboratories) for 30 mins at room temperature. Sections for rabbit and mouse antibodies were then washed five times with TBS-T for 3 mins each and sections for goat antibodies were washed three times with TBS-T for 5 mins each. The sections were then incubated with substrate chromogen, liquid 3,3’-diaminobenzidine (DAB) (DakoCytomation) at room temperature for 10 mins. Sections were washed five times in distilled water for 2 mins each before counterstaining with Mayer’s hematoxylin (Sigma-Aldrich) for 4 mins. Sections were then coverslipped using organic mounting media DPX (Asia Pacific Specialty Chemicals, Australia). Sections were imaged on an Olympus BX60 microscope (Olympus, Hamburg, Germany) with manual light exposure and ‘one push’ white balance on a background region. Images were then taken using an attached DP71 camera (Olympus). At least 15 - 20 images of the bronchial mucosa, including the basement membrane and epithelial layer, were taken per section at X20 magnification.

Quantification of immunostaining of the images taken was completed using Image Pro Plus version 7 Software (MediaCybernetics) to quantify the area of staining (the number of brown pixels in the image) [[41](#_ENREF_41), [42](#_ENREF_42)]. Area of interest analysis was done for the evaluation of epithelial specific immunostaining of CTSD and 2 patients were excluded due to excessive epithelial layer desquamation. Data points greater than 2 standard deviations away from the mean were excluded as outliers.
